# One major facilitator superfamily transporter is responsible for propionic acid tolerance in *Pseudomonas putida* KT2440

**DOI:** 10.1111/1751-7915.13597

**Published:** 2020-05-31

**Authors:** Chao Ma, Qingxuan Mu, Yubin Xue, Yanfen Xue, Bo Yu, Yanhe Ma

**Affiliations:** ^1^ CAS Key Laboratory of Microbial Physiological & Metabolic Engineering State Key Laboratory of Mycology Institute of Microbiology Chinese Academy of Sciences Beijing 100101 China; ^2^ State Key Laboratory of Microbial Resources Institute of Microbiology Chinese Academy of Sciences Beijing 100101 China; ^3^ University of Chinese Academy of Sciences Beijing 100049 China

## Abstract

Propionic acid (PA) has been widely used as a food preservative and chemical intermediate in the agricultural and pharmaceutical industries. Environmental and friendly biotechnological production of PA from biomass has been considered as an alternative to the traditional petrochemical route. However, because PA is a strong inhibitor of cell growth, the biotechnological host should be not only able to produce the compound but the host should be robust. In this study, we identified key PA tolerance factors in *Pseudomonas putida* KT2440 strain by comparative transcriptional analysis in the presence or absence of PA stress. The identified major facilitator superfamily (MFS) transporter gene cluster of PP_1271, PP_1272 and PP_1273 was experimentally verified to be involved in PA tolerance in *P. putida* strains. Overexpression of this cluster improved tolerance to PA in a PA producing strain, what is useful to further engineer this robust platform not only for PA synthesis but for the production of other weak acids.

## Graphical abstract

In this study, one major facilitator superfamily transporter (MFS2, locus tags of PP_1271‐PP_1273), but not MFS1 (locus tag of PP_0503), is beneficial to propionate tolerance in *Pseudomonas putida* strain KT2440. Thus, this study revealed the key genes responsible for PA tolerance in *P. putida* strains and provided the useful information to further engineer robust strains for PA production.

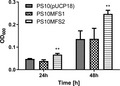

## Introduction

Propionic acid (PA) is a valuable C3 platform chemical, which is widely used in the agricultural and pharmaceutical industries. Currently, the most used commercial route to produce PA uses petrochemical methods (Zhang and Yang, 2009). However, the harmful effects of pollution from the use of unsustainable fossil fuels as starting substrate for the production of chemicals, have led researchers to seek alternative routes, such as using renewable bioresources by microbial cell factories (Ramos and Duque, 2019). To date, biological PA production is mainly achieved by fermentation using *Propionibacteria* under anaerobic conditions (Suwannakham and Yang, 2005; Zhang and Yang, 2009; Zhuge *et al*., 2015). However, PA production in *Propionibacteria* is constrained by slow growth rates and high costs of product purification since by‐products, such as acetate and succinate, are tightly coupled with PA fermentation (Kandasamy *et al*., 2013). In addition, PA itself is a strong growth inhibitor and is capable of reducing the *Propionibacterium* growth rate by 50% at a concentration as low as 1% (w/v) (Blanc and Goma, 1987). Thus, other hosts and alternative pathways for PA production need to be explored.

l‐Threonine is an inexpensive amino acid produced by biological fermentation of starch, which is commercially available in bulk quantities. Gonzalez‐Garcia *et al*. (2017) proposed that the L‐threonine degradation pathway may be a feasible and energetically efficient pathway for PA production. While the *E. coli* pathway yields formate (Hesslinger *et al*., 1998), *Pseudomonas putida* KT2440 uses a pathway that produces non‐toxic CO_2_ (Ma *et al*., 2020). Additionally, *P. putida* KT2440 is a safe‐certified strain (Kampers *et al*., 2019) and is already used in numerous biotechnological applications (Poblete‐Castro *et al*., 2012; Calero and Nikel, 2019). *Pseudomonas putida* is also a highly robust species known for having a higher stress tolerance compared with most *E. coli* strains (Calero *et al*., 2018). In a previous study, we developed an efficient cell factory to produce highly pure PA by engineering the L‐threonine degradation pathway in *P. putida* KT2440 (Ma *et al*., 2020).

The inhibitory effect of PA on microbial growth poses a big challenge for its efficient microbial production (Abbott *et al*., 2007). Thus, other than engineering an efficient cell factory for this transformation process, it is also necessary to improve the microbial resistance to PA. In this study, we identified a major facilitator superfamily (MFS) transporters in *P. putida* KT2440. The overexpression of the specific MFS genes under a constitutive promoter enhanced the growth rate in the presence of PA in the medium as well as the PA production rate. This study provides new insights into PA stress responses in *P. putida* strains and lays the foundation for developing robust strains for PA production.

## Results and discussion

### Knockout of PA degradation pathway decreases PA tolerance capacities in *P. putida*

**Fig. 1:**
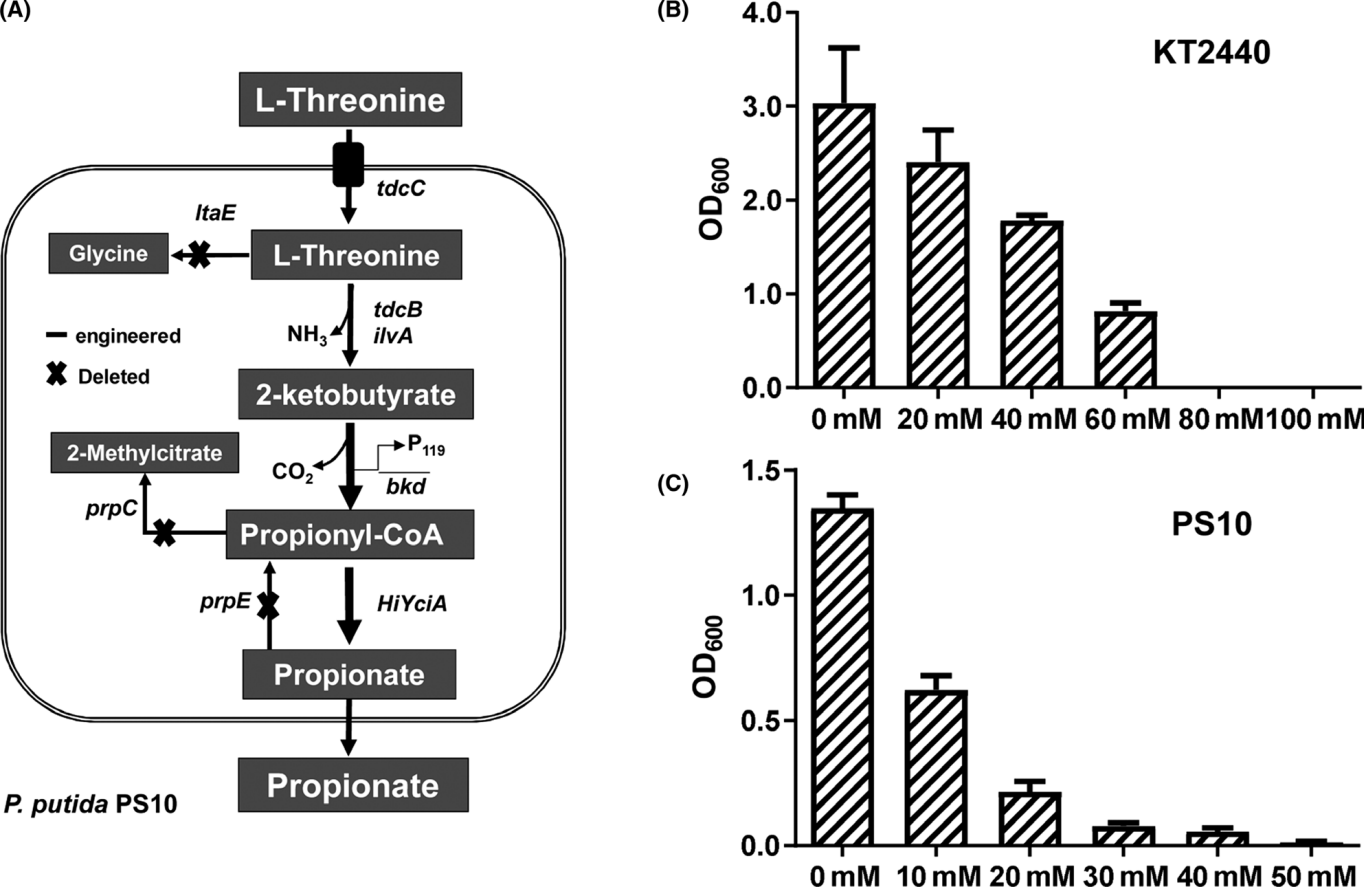
Propionate tolerance capacities of *P. putida* strains. A. The genetic background of engineered strain PS10. B. The cell growth of native strain *P. putida* KT2440 in M9 medium with different concentrations of propionate (from 0 to 100 mM). C. The cell growth of engineered strain *P. putida* PS10 with different concentrations of propionate (from 0 to 50 mM). The figure shows the mean values of three independent assays. The standard deviation is represented by *error bars*. *lta*E, l‐threonine aldolase gene; *prp*C, methylcitrate synthase gene; *prp*E, propionyl‐CoA synthase gene; *tdcB* and *ilvA*, l‐threonine deaminase genes from *E. coli*; *tdcC*, l‐threonine permease from *E. coli*; *HiYciA*, acyl‐CoA thioesterase from *Haemophilus influenza* (the codon‐optimized sequence was deposited in NCBI database under the accession number of MN536675); *bkd*, the endogenous branched‐chain alpha‐keto acid dehydrogenase complex gene and P_119_, the strong J23119 promoter. All *E. coli* gene sequences could be referenced from NCBI database under the accession number of NC_000913.3.

*Pseudomonas putida* PS10 is an engineered strain that transforms l‐threonine to PA with high efficiency (Ma *et al*., 2020). To test the initial tolerance of *P. putida* strains, the native strain KT2440 and the strain PS10 were first cultivated in medium with different concentrations of PA. As shown in Figure 1, the growth was significantly inhibited with the increase in PA concentration, especially in the PS10 strain. The OD_600_ value of KT2440 reached values of 1.0 in the presence of 60 mM of PA in the medium, while PS10 growth was almost completely inhibited above 30 mM of PA. Moreover, 10 mM PA reduced PS10 growth by more than 50%. PS10 had been engineered to favour PA production through the removal of all the genes involved in PA metabolic pathways (Fig. 1), such as the genes of methylcitrate synthase (PP_2335, *prpC*), propionyl‐CoA synthase (PP_2351, *prpE*) and L‐threonine aldolase (PP_0321, *ltaE*; Ma *et al*., 2020). Knock‐out of enzymes responsible for the transformation of intermediates in the PA production pathway led to an increased sensitivity to PA, most likely because the cells accumulate higher intracellular concentrations of PA. A recent study also reported that in *Yarrowia lipolytica*, the deletion of a gene in the methylcitrate pathway causes a severe growth defect (Park and Nicaud, 2020). A useful chassis for production should be endowed with suitable production properties but also the required determinants for tolerating the product. Despite the PS10 strain being engineered to accumulate high amounts of PA, exploration of PA tolerance with *P. putida* strain is still desirable.

### Comparative transcriptome analysis in the presence and absence of PA stress

Enhancing the cellular tolerance to toxic products has been a major issue for many bioprocesses. Previously, some studies aimed to improve PA tolerance in yeast by adaptive laboratory evolution, as listed in the literature (Xu *et al*., 2019). However, to date, no studies have focused on the mechanisms underlying PA tolerance in *Pseudomonas*. In this study, we performed a comparative transcriptional analysis in the presence and absence of 10 mM PA to identify key genes involved in PA tolerance. Since PS10 cells cultured in the presence of PA had a longer lag phase and a slower growth rate, we collected the cells after 20 h (OD_600_ value = 0.57), whereas cells cultured without PA were collected after 16 h (OD_600_ value = 1.31). Total RNA from three independent replicates was extracted and then subjected to analysis. As expected, we found a large number of upregulated genes involved in cell metabolism and stress regulation in the cells cultured with PA (data not shown). A recent study showed that overexpression of the *MFS1* gene could improve PA tolerance in *Yarrowia lipolytica*, a lipid producer that uses PA as the sole substrate (Park and Nicaud, 2020). Thus, we searched for MFS transporter genes differentially expressed in *P. putida* in the presence of PA. We found 7 MFS genes annotated in the KT2440 genome showing differential expression (Table 1). Among these, PP_0503, PP_1271, PP_1272 and PP_1273 displayed an up‐regulated pattern.

**Table 1:** The differential transcription of MFS genes in *P. putida* PS10 grown with or without propionate.

Locus tags	log2FoldChange^a^	*P*_val_ ^b^	*P*_adj_ ^c^	Description
PP_0503	1.6301	3.93E−12	1.27E−10	MFS transporter
PP_1271	0.80337	0.0016205	0.0094414	Putative Multidrug efflux MFS transporter
PP_1272	1.3797	0.0013621	0.0082056	Multidrug MFS transporter membrane fusion protein
PP_1273	0.67662	0.0079972	0.034623	Multidrug MFS transporter outer membrane protein
PP_2241	−0.90346	0.00021105	0.0016756	MFS transporter
PP_4758	−0.7066	0.010584	0.042964	MFS transporter
PP_5368	−0.98477	0.00063549	0.0042786	MFS transporter

^a^ log2foldchange represents the expression level change of PS10 cultivated with propionate as compared to that without propionate. The data showed the mean values of three replicates.

^b^ *P*_val_ = *P* value, which means the probability of an event or outcome in a statistical experiment.

^c^ *P*_adj_ = *q* value, which is the *p* value corrected by FDR (False Discovery Rates) method. The smaller the *q* value is, the more significant the difference is in gene expression.

### Validation of the role of MFS transporter genes on PA tolerance

**Fig. 2:**
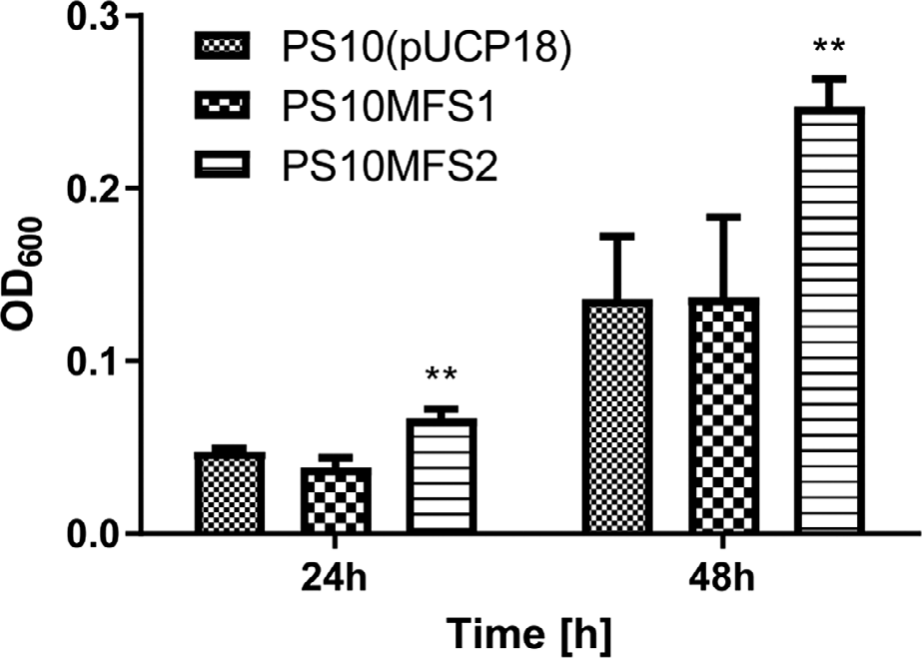
The growth of *P. putida* strains with different MFS transporter gene overexpression under propionate stressed condition respectively. Overnight cultures of *P. putida* strains were diluted in M9 medium containing 30 mM sodium propionate to give the initial optical density (OD_600_) of 0.01. Then, the strains were cultivated at 30°C with constant shaking for 48 h. Growth of the control strain PS10(pUCP18) with the empty vector of pUCP18, the PS10MFS1 strain (PS10 harbouring MFS transporter gene PP_0503), and the PS10MFS2 strain (PS10 harbouring MFS transporter gene cluster of PP_1271, PP_1272 and PP_1273) were monitored at 24 h and 48 h respectively. The figure shows the mean values of three independent assays. The standard deviation is represented by *error bars*. All t‐tests compare the cell growth of PS10MFS2 against PS10 (**P < 0.01).

The four induced genes mentioned above were then over‐expressed in strain PS10 to verify their ability to increase PA tolerance. The PP_0503 gene was cloned into the pUCP18 vector, and the resulting plasmid was called pMFS1. Since PP_1271, PP_1272 and PP_1273 were clustered on the chromosome, we cloned the entire operon into the pUCP18 to generate the plasmid of pMSF2. All the genes were driven by the *lac* promoter in the plasmids, which serves as a constitutive promoter in *P. putida* (Elmore *et al*., 2017). The strains transformed with pMSF1 and pMSF2 were called PS10MFS1 and PS10MFS2 respectively. To assess the PA tolerance, we compared the growth of PS10MFS1 and PS10MFS2 with that of a control PS10 strain transformed with the empty pUCP18 vector (PS10(pUCP18); Fig. 2). In the presence of 30 mM sodium propionate, the growth of PS10MFS1 and PS10(pUCP18) was comparable, both at 24 h and 48 h, suggesting that the PP_0503 gene did not contribute to PA tolerance in these experimental conditions. In contrast, the PS10MFS2 strain displayed a greater PA tolerance compared with that of the PS10(pUCP18) strain. Its final OD_600_ value was almost twice than that of PS10(pUCP18). This result suggests that the PP_1271, PP_1272 and PP_1273 gene cluster may be involved in PA tolerance in *P. putida*.

### Overexpression of the MFS transporter genes improved PA production

**Fig. 3:**
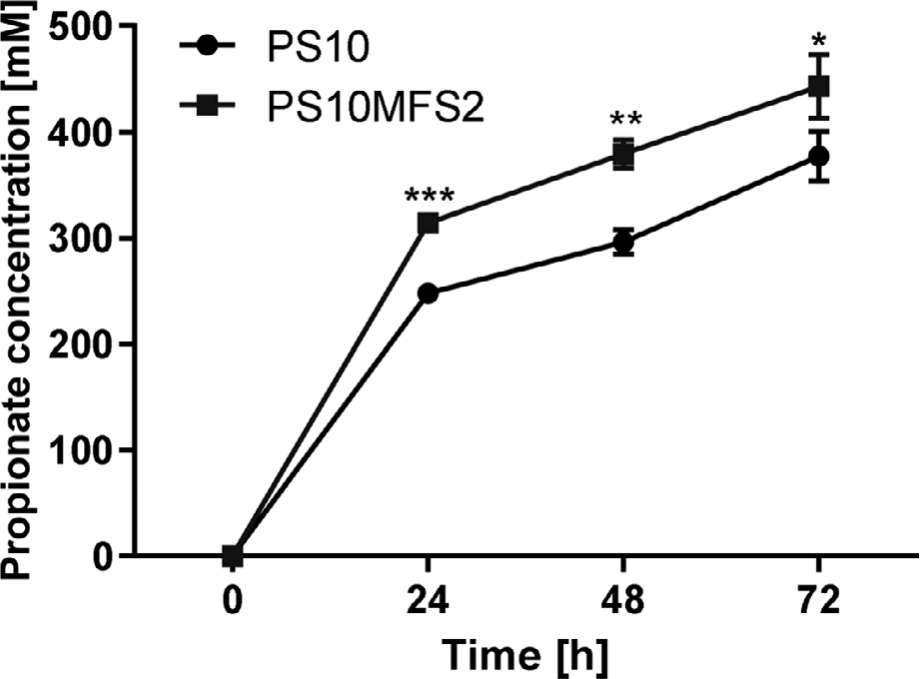
Bioconversion assays of propionate production from 600 mM l‐threonine. The biomass concentrations were all set at OD_600_ value of 20. Propionate production by the PS10 strain (●) and the PS10MFS2 strain (PS10 harbouring MFS transporter gene cluster of PP_1271, PP_1272 and PP_1273, ■). The figure shows the mean values of three independent assays. The standard deviation is represented by *error bars*. All t‐tests compare the PA production by PS10MFS2 against PS10 (***P < 0.001, **P < 0.01 and *P < 0.05).

Along with testing the PA tolerance, we also investigated whether the overproduction of an inducible MFS transporter was associated with an increased PA production. We first tested biomass with an OD_600_ = 30 supplemented with an L‐threonine substrate at a concentration of 500 mM. Under these conditions, after 24 h the PS10 strain produced 464.5 ± 2.5 mM of PA, while the PS10MFS2 strain produced slightly more PA (488.8 ± 4.1 mM). This difference was statistically significant (*P* value < 0.01). Since PA production was performed by resting cells with high density, we managed to obtain a concentration of PA more than 10‐fold higher than the tolerance level. Next, we increased the l‐threonine concentration to 600 mM using a lower biomass level (OD_600_ = 20). After 72 h, the PS10 strain produced 376.8 mM of PA, significantly lower than that of the PS10MFS2 strain, which produced 442.9 mM of PA (*P* value < 0.05; Fig. 3). For both strains, the PA yield was lower compared with that obtained using 500 mM of the substrate, but this depended on the lower biomass used for this experiment. However, also in these experimental conditions, the PS10MFS2 strain showed higher productivity than that of the PS10 strain throughout the whole process. This result further confirms that the MSF2 gene cluster has an important role in regulating *P. putida* PA tolerance mechanisms. Notably, although the overexpression of the MFS2 transporter gene cluster showed increased PA tolerance and production, the PS10Δ(1271‐1273) strain, in which the cluster was deleted, showed a production performance (451.8 ± 8.8 mM) comparable to that of the parental PS10 strain at 24 h. This suggests that in *P. putida* strain derived from KT2440, PA tolerance does not rely solely on the MFS2 cluster; thus, in the absence of the cluster, other enzymes may regulate PA tolerance.

Propionic acid is used as a food preservative due to its potent inhibitory effect on microbial growth. Unfortunately, this characteristic also poses a big challenge for its high‐efficiency microbial production. *Propionibacterium acidipropionici* strains with tolerance to PA were obtained using a time‐consuming adaptive evolution approach (Suwannakham and Yang, 2005). Compared to the parental strain, the adapted *P. acidipropionici* had a longer rod morphology and higher H^+^‐ATPase activity (Zhang and Yang, 2009). However, little is known on the genetic control circuits related to PA stress. Recently, Xu *et al*. (2019) reported potassium influx to be a PA tolerance mechanism in *Saccharomyces cerevisiae*. The unique mutation in the *TRK1* gene, which encodes a high‐affinity potassium transporter, was proven to be critical for tolerance to PA in yeast.

During evolution, microorganisms have evolved different mechanisms, such as membrane efflux pumps, to eliminate toxic substances (Ramos *et al*., 2002). The presence of multiple pumps explains the unusually high tolerance observed in *P. putida* towards several organic solvents and antibiotics (Molina‐Santiago *et al*., 2014; Cuenca Mdel *et al*., 2016). Efflux pumps increase bacterial tolerance toward toxic substances by removing them from the cell bypassing the periplasm (Basler *et al*., 2018). The overexpression of efflux pumps to increase the tolerance of bacterial strains has been extensively described in the literature. However, very few studies have investigated PA tolerance, compared with the tolerance against other weak acids, such as acetic and lactic acids. MFS transporters facilitate the movement of various substrates, including ions, sugar phosphates, drugs, amino acids and peptides, across the cytoplasmic and internal membranes (dos Santos *et al*., 2014). Recently, one MFS transporter gene (*MFS1*) has been correlated to PA tolerance in *Y. lipolytica*, a bacterium that could use propionate as the substrate for lipid production (Park and Nicaud, 2020). Overexpression of *MFS1* in *Y. lipolytica* caused a greater tolerance to PA. To date, no study on the PA tolerance of *P. putida* strains has been reported, especially in the context of PA production. In this study, the overexpression of the MFS transporter gene cluster of PP_1271, PP_1272 and PP_1273 increased PA tolerance in *P. putida*. Notably, the same cluster was found to be associated with *p*‐hydroxybenzoate export in the *P. putida* S12 strain (Verhoef *et al*., 2010), and upregulated in response to toluene and phenol stress (Volkers *et al*., 2009; Verhoef *et al*., 2010). Additionally, the PP_1271 gene was reported to be involved in the urea export in the *P. putida* KT2440 strain (Reva *et al*., 2006). These findings further confirm that MFS transporters exhibit a wide spectrum of substrate specificity (Pasqua *et al*., 2019). In addition, it has been shown that MFS transporters influence stress response molecular machinery and control the membrane potential and/or the internal pH (dos Santos *et al*., 2014). In our engineered biotransformation reaction, one mol of L‐threonine was transformed to one mol of propionate, with equal mol of NH_3_ and CO_2_ (Ma *et al*., 2020). Thus, the released NH_3_ may neutralize PA *in vivo*. Although the mechanism of the MFS transporter locus tags of PP_1271‐PP_1273 in the stress response to PA remains unclear in this study, it is most likely related to the transport of PA outside the cells, since the pH of the broth remained neutral during the biotransformation process (Ma *et al*., 2020).

## Conclusions

The soil bacterium *P. putida* KT2440 is gaining increasing biotechnological interest due to its ability to tolerate different types of stress (Calero *et al*., 2018). The strain has been engineered for ethanol biosynthesis (Nikel and de Lorenzo, 2014) and the efficient production of vanillin from ferulic acid (Graf and Altenbuchner, 2014). Moreover, the strain also showed great potential as a platform for the synthesis of aromatic compounds, such as l‐phenylalanine, 2‐phenylethanol and *trans*‐cinnamate (Molina‐Santiago *et al*., 2016). Recently, a thoroughly genome‐scale metabolic modelling of KT2440 has been published, confirming its broad metabolic capabilities (Nogales *et al*., 2020). However, further engineering efforts to increase the robustness of the strain are vital for transforming *P. putida* to the workhorse in the bio‐industry (Nikel and de Lorenzo, 2018). For instance, the ABC transporter Ttg2ABC and the cytochrome c maturation system (ccm) were reported to play an important role in the tolerance to *p*‐coumaric acid in the KT2440 strain (Calero *et al*., 2018). We successfully identified an MFS transporter gene cluster (MFS2) comprising three genes, PP_1271, PP_1272 and PP_1273, which regulated the PA tolerance in *P. putida* in this study. Notably, MFS transporters usually exhibit a large spectrum of substrate specificity. Previously, the MFS2 cluster has been associated with the export of toluene, phenol, *p*‐hydroxybenzoate and urea. Thus, our study lays the foundation for future improvement efforts of *P. putida* as a platform for the production of other short‐chain carbonic acids other than PA.

## Conflict of interest

No declared.

## Supporting information


**Appendix S1** **.** The detailed information of experiment procedures was provided, including cultivation conditions, genetic manipulation methods, primers for amplification and cloning procedures, strains & plasmids used in this study, as well as the process for PA production and the HPLC analysis methods.
