# Supplementary material for: One major facilitator superfamily transporter is responsible for propionic acid tolerance in Pseudomonas putida KT2440
Source: Microb Biotechnol. 2020 May 31;14(2):386–91. doi: 10.1111/1751-7915.13597 (PMC7936288; doi:10.1111/1751-7915.13597)
Supplement: Supplementary file 1 — Appendix S1 . The detailed information of experiment procedures was provided, including cultivation conditions, genetic manipulation methods, primers for amplification and cloning procedures, strains & plasmids used in this study, as well as the process for PA production and the HPLC analysis methods. [file MBT2-14-386-s001.docx]

Supplementary file

**One major facilitator superfamily transporter is responsible for propionic acid tolerance in *Pseudomonas putida* KT2440**

Chao Ma^1,2,3^, Qingxuan Mu^1,3^, Yubin Xue^1,3^, Yanfen Xue^2^, Bo Yu^1*^, Yanhe Ma^2**^

*^1^ CAS Key Laboratory of Microbial Physiological & Metabolic Engineering, Institute of Microbiology, Chinese Academy of Sciences, Beijing 100101, China*

*^2^ State Key Laboratory of Microbial Resources, Institute of Microbiology, Chinese Academy of Sciences, Beijing 100101, China*

*^3^ University of Chinese Academy of Sciences, Beijing 100049, China*

For correspondence:

^*^E-mail: yub@im.ac.cn; Tel./Fax +86-10-64806132

^**^E-mail: [mayanhe@im.ac.cn](mailto:mayanhe@im.ac.cn); Tel./Fax +86-10-64807616

**Experimental procedures**

*Strains and cultivation conditions*

*E. coli* and *P. putida* strains were grown in Luria-Bertani (LB) medium (10 g/L tryptone, 5 g/L yeast extract, and 10 g/L NaCl) for all DNA manipulations. For selection of plasmids 50 μg/ml gentamicin (Gm) was added. For PA tolerance tests, M9 minimal medium (17.16 g/L Na_2_HPO_4_·12 H_2_O, 3 g/L KH_2_PO_4_, 0.5 g/L NaCl, 1 g/L NH_4_Cl) with different concentrations of PA were used for growth of *P. putida* strains, supplemented with 1% glucose, 2 mM MgSO_4_, 0.1 mM CaCl_2_ and 50 μg/ml Gm. Overnight cultures of the *P. putida* strains were diluted in M9 medium to give the initial turbidity of 0.01 under the wavelength of 600 nm. Then the strains were cultivated at 30°C with constant shaking for 48 hours. Growth was monitored by measuring the turbidity values.

*Gene cloning and plasmid construction*

*E. coli* DH5α (TransGen Biotech, Beijing, China) was used for plasmid construction. *E. coli* strains were transformed with plasmid DNA via Calcium-dependent transformation (Mandel and Higa, 1970). *P. putida* strains were transformed with plasmid DNA via electroporation (Cho et al., 1995). The strains and recombinant plasmids used in this study are listed in Table S1. The PP_0503 gene and PP_1271-1273 gene cluster were PCR-amplified from the genome of *Pseudomonas putida* KT2440 according to the genome sequence with NCBI accession number of NC_002947.4. The primers used for plasmid construction were listed in Table S2. These genes were inserted into pUCP18 at the sites of ScaI and XmaI via Gibson assembly (Casini et al., 2014).

*KT2440 markerless gene deletion*

For chromosomal deletions in *P. putida* KT2440, the *sacB* counterselection system was used as described previously (Schweizer and Hoang, 1995). First, the up- and downstream regions of the target gene were PCR-amplified using chromosomal DNA of *P. putida* KT2440 as template. These fragments were cloned via Gibson assembly into pK18Gm. All recombinant plasmids were validated by nucleotide sequencing. *E. coli* S17.1 (Simon et al. 1983) was transformed with recombinant plasmid via electroporation. Overnight cultures of *E. coli* S17.1 with plasmid and *P. putida* KT2440 grown in LB with and without gentamicin, respectively, were mixed equally (200 μl each) and 100 μl of that mixture was dropped onto a LB agar plate without antibiotics. After incubation for 12 h at 37 °C, grown cells were scraped off the plate into the other LB agar plate containing 50 μM gentamicin and 25 μM irgasan (for counterselection of the *E. coli* donor). After incubation for 24 h at 37 °C, the colonies, which had done the first homologous recombination, were toothpicked on a LB agar plate with 25 μM irgasan and 20% sucrose. After incubation for 24 h at 37 °C, the colonies, which had done the second homologous recombination, were toothpicked on a LB agar plate with and without gentamicin, respectively. The colonies, which didn’t grow on LB agar plates containing 50 μM gentamicin, were validated by PCR and nucleotide sequencing.

*The process for PA production*

Overnight cultures of *P. putida* strains were diluted 1:100 in fresh LB medium and grown for 12 h at 30 °C in shaking flasks (200 rpm). Cells were harvested by centrifugation (10 min, 3,500 × g, room temperature), washed, and resuspended with 50 mM phosphate buffered saline (8.0 g/L NaCl, 0.2 g/L KCl, 2.9 g/L Na_2_HPO_4_∙12 H_2_O, 0.24 g/L KH_2_PO_4_) to give the final optical density (OD_600_) of 20 or 30. The bioconversion for testing the performance of different strains was conducted with a reaction volume of 5 ml in 50 ml Erlenmeyer flasks at 37 °C under shaking conditions (200 rpm). Samples of 200 μL were taken after several hours of conversion time and subjected to analysis of PA titers with high performance liquid chromatography (Suwannakham and Yang, 2005; Zhang *et al*., 2016).

*Analytical methods*

The samples were detected by high performance liquid chromatography (HPLC) (Agilent 1260 series, Germany) equipped with an Aminex HPX-87H 300 mm×7.8 mm column (Bio-Rad) and a diode array detector at 210 nm. Analysis was performed with the mobile phase of 18.0 mM H_2_SO_4_ at a flow rate of 0.6 mL/min at 35 °C for 30 min. The injection volume was 10 μL. The analytical method was experimentally verified with standard PA and the retention time is about 18 min. The concentrations of standard PA included 5 mM, 10 mM, 20 mM, 50 mM, 100 mM and 400 mM were applied to make the calibration curve. The concentrations in samples were quantitatively determined with the calibration curve using linear regression. As the biotransformation was conducted by the resting cells in the phosphate buffer, no other impurities are in the broth. The HPLC peak signal of PA is specific and no other organic acids, such as formate, acetate, lactate, succinate as well as substrate L-threonine, have the same retention time with PA under the above analytical conditions.

**Table S1** Strains and plasmids used in this study.

| Strain or plasmid | Description | Reference or source |
| --- | --- | --- |
| Strains  *E. coli* DH5α  *E. coli* S17.1  *P. putida* PS10  *P. putida* PS10MFS1  *P. putida* PS10MFS2  *P. putida* PS10Δ(1271-1273)  Plasmids  pUCP18  pK18Gm  pMFS1  pMFS2  pK18-Δ(1271-1273) | Strain used for general cloning  *recA pro hsdR* RP4-2*-Tc*::*Mu-Km*::Tn7  KT2440Δ*prpC*::P_lac_-*tdcBC*, Δ*ltaE*::P_lac_-*ilvA*, Δ*bkdR*::P_119_-*bkd*, Δ*lacI*::P_lac_-*HiYciA*, Δ*prpE*  PS10 harboring pMFS1  PS10 harboring pMFS2  PS10Δ(PP_1271, PP_1272 and PP_1273)  *P. putida* expression vector, Gm^R^, P_lac_  Suicide vector, *sacB*, Gm^R^  pUCP18 harboring PP_0503 gene  pUCP18 harboring PP_1271, PP_1272 and PP_1273 gene cluster  pK18Gm with 500bp upstream region and 500bp downstream region of PP-1271-1273 cluster | TransGen Biotech Co. Ltd, China  Simon *et al.* (1983)  Lab stock  This study  This study  This study  Schweizer *et al*. (1991)  Quenee *et al.* (2005)  This study  This study  This study |

P_lac_, *lac* promoter; Gm^R^, gentamicin resistance

**Table S2** Primers used in this study.

| **Primer** | **Sequence (5'––3')** | **Purpose** |
| --- | --- | --- |
| 18-0503-F | caatttcacacaggaaacagctatgaccatgacaccttcgctgacacgctggataac | Cloning genes inserted into pUCP18 |
| 0503-18-R | ctgcaggtcgactctagaggatccccgggctacaagttctgacctgaacattgcaggg |  |
| 18-1271-F | caatttcacacaggaaacagctatgaccATGACTTCCCTGACGGCGCCCTCTGCGG |  |
| 1273-18-R | ctgcaggtcgactctagaggatccccgggCTAACCGTGAGCATTCGGCGGCGGCTCATC |  |
| 18-1271up-F | gattacgaattcgagctcggtacccgggGTGGGCCACCCTATCTTCGATGCCCCGATC | PP_1271, PP_1272 and PP_1273 markerless deletion |
| 1271up-down-R | CGCCCTCCAGATACTGCGCGTCCTCTGAGAATACATCTCCAGCAACAATTTC |  |
| 1271up-down-F | GAAATTGTTGCTGGAGATGTATTCTCAGAGGACGCGCAGTATCTGGAGGGCG |  |
| 1273down-18-R | gtaaaacgacggccagtgccaagcttCTGGCGGCAGACTCGGCGTGGTGGCCTTTG |  |

**References**

Casini, A., MacDonald, J.T., de Jonghe, J., Christodoulou, G., Freemont, P.S., Baldwin, G.S., *et al.* (2014) One-pot DNA construction for synthetic biology: the modular overlap-directed assembly with linkers (MODAL) strategy. *Nucleic Acids Res* **42:** e7.

Cho, J.H., Kim, E.K., and So, J.S. (1995) Improved transformation of *Pseudomonas putida* KT2440 by electroporation. *Biotechnol Tech* **9:** 41-44.

Mandel, M., and Higa, A. (1970) Calcium-dependent bacteriophage DNA infection. *J. Mol Biol.* **53:**159-162.

Schweizer, H.P. (1991) *Escherichia*-*Pseudomonas* shuttle vectors derived from pUC18/19. *Gene* **97:** 109-112.

Schweizer, H.P., and Hoang, T.T. (1995) An improved system for gene replacement and *xyl*E fusion analysis in *Pseudomonas aeruginosa*. *Gene* **158:** 15-22.

Suwannakham, S., and Yang, S.T. (2005) Enhanced propionic acid fermentation by *Propionibacterium acidipropionici* mutant obtained by adaptation in a fibrous-bed bioreactor. *Biotechnol Bioeng* **91:** 325-337.

Simon, R., Priefer, U., and Puhler, A. (1983) A broad host range mobilization system for *in vivo* genetic engineering: transposon mutagenesis in gram negative bacteria. *Bio-Technology* **1:** 784-791.

Quenee, L., Lamotte, D., and Polack, B. (2005) Combined sacB-based negative selection and cre-lox antibiotic marker recycling for efficient gene deletion in *Pseudomonas aeruginosa*. *Biotechniques* **38:** 63-67.

Zhang, C., Qi, J., Li, Y., Fan, X., Xu, Q., Chen, N., and Xie, X. (2016) Production of alpha-ketobutyrate using engineered *Escherichia coli* via temperature shift. *Biotechnol Bioeng* **113:** 2054-2059.
